# Comparative assessment of periodontal health among adolescents in Rabat, Morocco using AAP/EFP 2017 and CDC/AAP 2012 classifications

**DOI:** 10.1038/s41598-025-31391-6

**Published:** 2025-12-09

**Authors:** Brahim Jabri, Mohamed Achmit, Slimane Faouzi, Samira Hsaine, Hanane Jamai-Nafia, Soumia Ait-ou-amar, Oumkeltoum Ennibi

**Affiliations:** 1 Research laboratory: care, health and environment, High Institute of Nursing Professions and Health Technics, Rabat, Morocco; 2 Research laboratory in oral biology and biotechnology, Faculty of dental medicine, Mohammed V University, Rabat, Morocco; 3 High Institute of Nursing Professions and Health Technics, Casablanca, Morocco; 4 UR Microbiology, Biomolecules and Biotechnology Laboratory of Physical Chemistry and Biotechnologies of Biomolecules and Materials, Faculty of Sciences and Technology, Mohammedia, Morocco; 5 Laboratory of Natural Resources and Sustainable Development, Faculty of Sciences, Ibn Tofail University, Kenitra, Morocco; 6 Department of Periodontology, Faculty of Dental Medicine, Mohammed V University, Rabat, Morocco

**Keywords:** Gingivitis, Periodontitis, Adolescents, AAP classification, Oral health, Diseases, Medical research

## Abstract

Periodontal diseases are highly prevalent among adolescents and represent a growing public health challenge worldwide. This study aimed to assess the prevalence and clinical profile of periodontal conditions among Moroccan adolescents and to analyze their association with socio-demographic and behavioral factors. A cross-sectional survey was conducted among 400 students aged 12 to 18 years enrolled in public secondary schools in Rabat, Morocco, using a two-stage cluster sampling method. Periodontal status was evaluated by trained examiners using standardized indices, including probing pocket depth, clinical attachment loss, plaque index, and bleeding on probing. The results revealed that nearly two-thirds of participants presented signs of gingivitis, while a significant proportion exhibited clinical features consistent with periodontitis. Periodontal conditions were more frequent among older adolescents and were associated with poor oral hygiene practices. The findings highlight the early onset and considerable burden of periodontal disease in Moroccan adolescents, underscoring the importance of integrating targeted prevention and early detection strategies into school-based oral health programs. These data provide essential insights for guiding public health policies to improve adolescent oral health and to reduce the long-term impact of periodontal disease in Morocco.

## Introduction

 Periodontal diseases are recognized by the World Health Organization (WHO) as one of the most prevalent non-communicable diseases globally, affecting nearly one billion people and representing a major public health concern^1^. The recent WHO Global Oral Health Status Report further emphasized the urgent need to strengthen prevention and universal health coverage for oral health by 2030^2^. Beyond their local impact on tooth-supporting tissues, periodontal diseases have been linked to systemic conditions such as diabetes, cardiovascular disease, and adverse pregnancy outcomes, underlining the importance of effective surveillance and early intervention strategies^3,4^.

Although most epidemiological data focus on adults, there is growing evidence that periodontal inflammation already affects adolescents and young adults. Gingivitis is almost universal at this age, while early forms of periodontitis are becoming increasingly common, particularly in certain geographical regions^5,6^. This situation is particularly relevant in Morocco, where recent national surveys have reported high prevalence rates of gingivitis and periodontitis among adolescents, with stage I and II lesions accounting for the majority of cases^7,8^. This study highlights the importance of focusing on younger populations, as early periodontal deterioration can compromise oral health and quality of life and predict disease progression in adulthood.

A major limitation in interpreting prevalence data across populations lies in the variability of case definitions. The CDC/AAP 2012 criteria have been widely used in epidemiological studies, but they only classify moderate and severe forms of periodontitis and do not recognize gingivitis as a diagnostic entity^3^. In contrast, the 2017 classification proposed by the American Academy of Periodontology (AAP) and the European Federation of Periodontology (EFP) introduced a paradigm shift by including staging and grading of periodontitis and explicitly recognizing gingivitis as a clinical category. In this framework, gingivitis is defined as the presence of bleeding on probing (BOP) at ≥ 10% of sites, with no clinical attachment loss and no probing pocket depth > 3 mm^9^. Comparative studies have shown that the 2017 AAP/EFP case definitions detect a higher prevalence of periodontal alterations than the 2012 CDC/AAP criteria, particularly among adolescents and young populations^4,6^.

Based on this evidence, we hypothesized that applying the 2017 AAP/EFP classification to adolescents would identify a greater burden of periodontal conditions, particularly gingivitis and early-stage periodontitis, compared to the CDC/AAP 2012 case definitions. Therefore, the objectives of this study were: to assess the prevalence and clinical characteristics of periodontal diseases among school-aged adolescents in Rabat, Morocco; and to compare case definitions according to the AAP/EFP 2017 and CDC/AAP 2012 systems in order to evaluate the applicability and sensitivity of the new classification in this younger population.

## Methods

### Sample size and sampling method

This cross-sectional study was conducted among 400 students aged 12 to 18 years, enrolled in public secondary schools located in urban areas of Rabat province, Morocco. The sample size was calculated to estimate prevalence with 95% confidence and a 5% margin of error. Assuming a conservative estimated prevalence of 50%, the minimum required sample size was 384; to account for possible non-response, 400 students were ultimately enrolled.

**Fig. 1:**
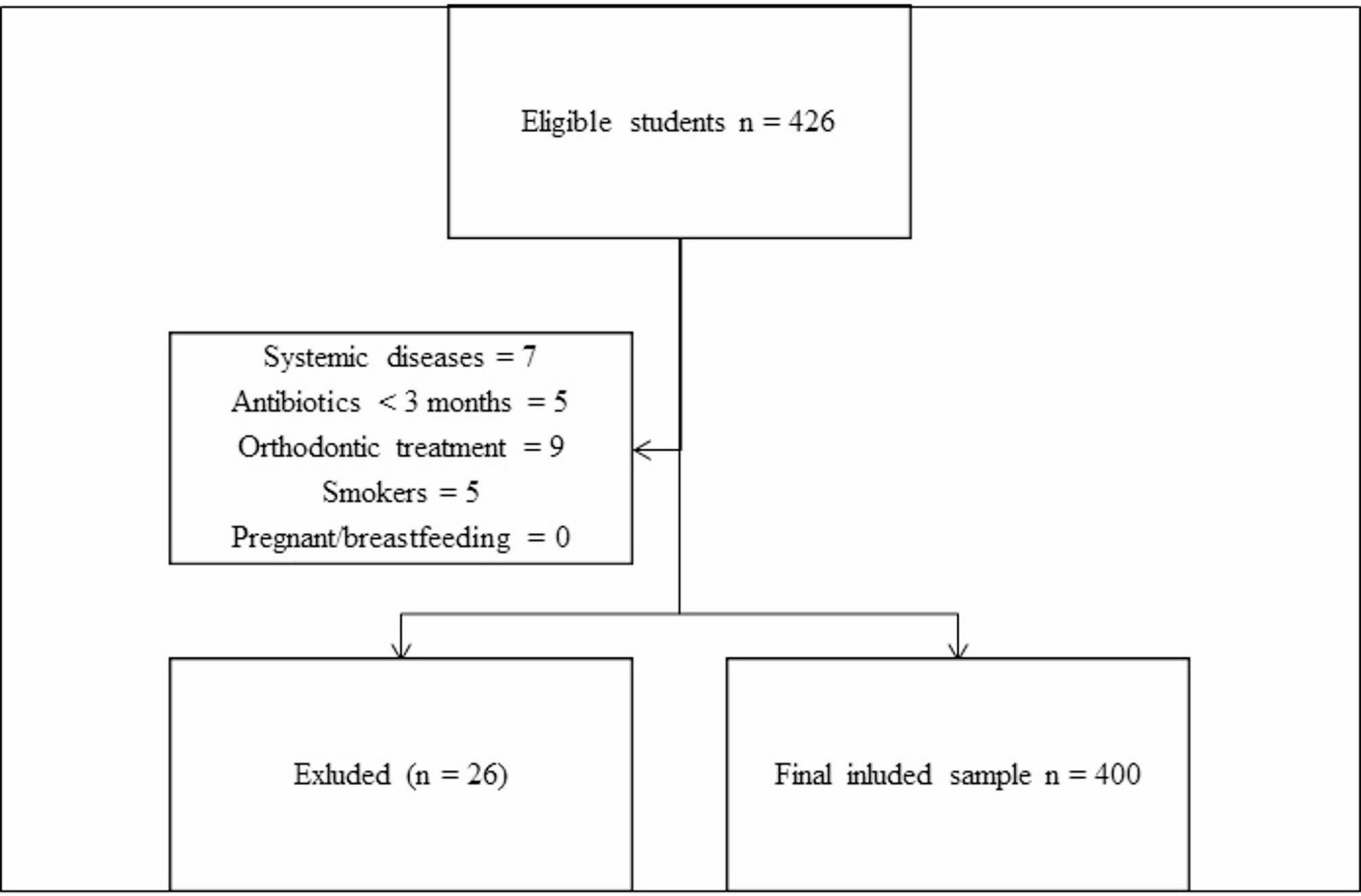
Flowchart of sampling procedures and study sample. The flow diagram shows the selection process of participants, including total students screened, exclusions, and final distribution into healthy, gingivitis, and periodontitis groups.

A two-stage cluster sampling method was used to ensure representativeness of adolescents in Rabat. In the first stage, public secondary schools were stratified by geographic sector (north, south, east, west, and center of the city). Within each stratum, schools were randomly selected proportionally to the number of students enrolled, in order to reflect the urban distribution of the adolescent population. In the second stage, classes were randomly chosen within each selected school, and all eligible students in those classes were invited to participate. This approach was designed to minimize selection bias and provide a representative sample of school-aged adolescents across Rabat (Fig. 1).

### Inclusion criteria

Eligible participants were school-aged adolescents (12–18 years) enrolled in public secondary schools in urban areas of Rabat, Morocco. All participants were required to be in good general health and to provide written informed consent signed by their parents or legal guardians, in accordance with ethical regulations regarding minors.

### Exclusion criteria

The following students were excluded:

- those with systemic diseases affecting the periodontium (e.g., diabetes, immunodeficiencies),
- those who had received antibiotic therapy within the three months preceding the survey,
- those undergoing orthodontic treatment,
- smokers (all forms of tobacco use),
- pregnant or breastfeeding females^10^.

### Clinical examination

All periodontal examinations were conducted by a single experienced periodontist using a UNC-15 probe. Measurements were taken at six sites per tooth, following a standardized protocol to ensure consistency. The recorded parameters included:

- Plaque Index (PI)^11^,
- Bleeding on Probing (BOP),
- Probing Pocket Depth (PPD),
- Clinical Attachment Level (CAL).

**Fig. 2:**
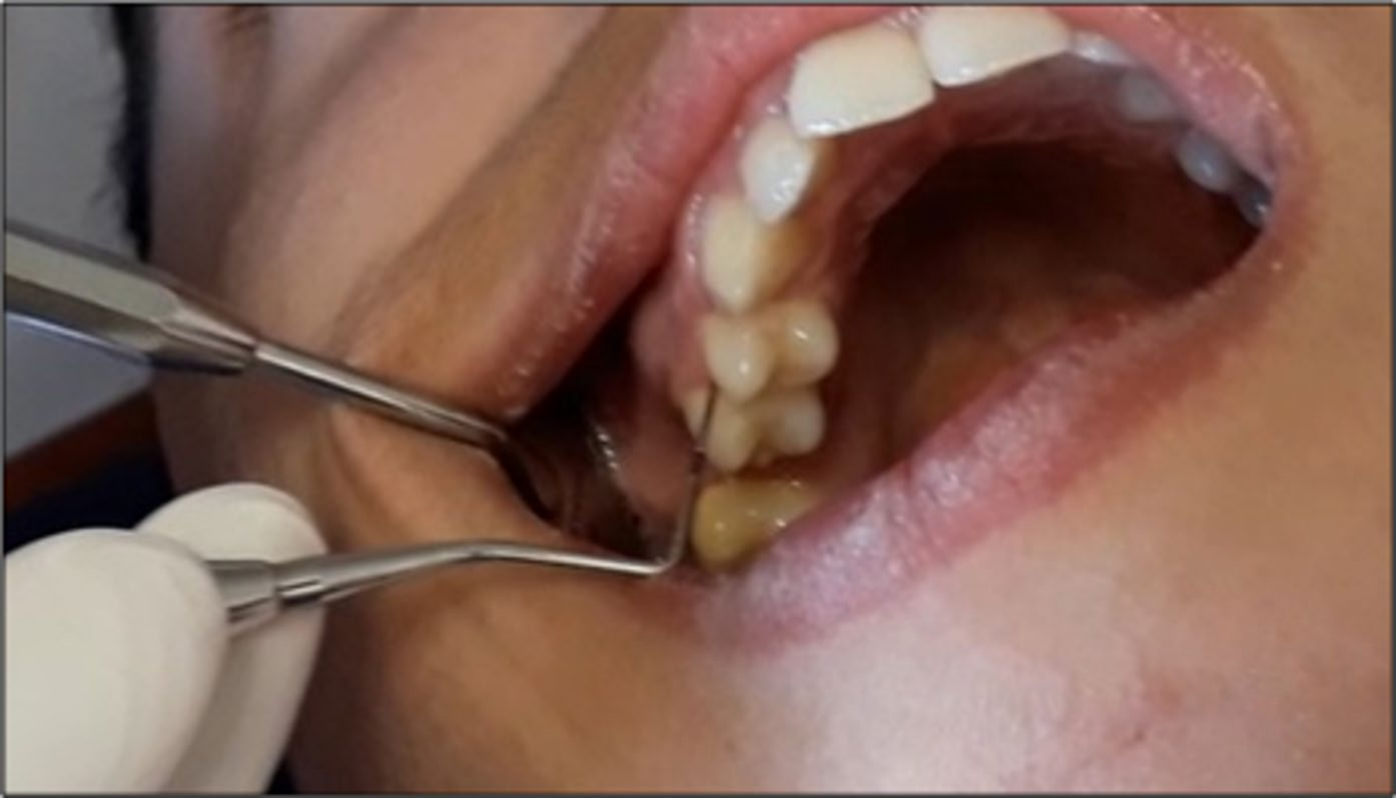
Photograph of the periodontal clinical examination. The image illustrates the method used to assess probing pocket depth (PPD), clinical attachment loss (CAL), bleeding on probing (BOP), and plaque index (PI).

Gingivitis was defined according to the 2017 World Workshop criteria^9^: presence of BOP at ≥ 10% of sites, with classification as localized (10–30%) or generalized (> 30%). Periodontitis was diagnosed when CAL was associated with pockets > 3 mm in at least two non-adjacent teeth, after excluding non-periodontal causes. Complementary periapical and bitewing radiographs were performed in adolescents clinically diagnosed with periodontitis to confirm bone loss and support staging according to the AAP/EFP 2017 criteria. Radiographic evaluation followed standard exposure and interpretation protocols to ensure diagnostic reliability (Fig. 2).

### Examiner reliability

No formal calibration session was conducted prior to the study, which is acknowledged as a limitation. However, to ensure measurement reproducibility, the examiner performed repeated probing measurements on 10 randomly selected students during the pilot phase. The repeated values were consistent within ± 0.5 mm, indicating good intra-examiner reliability. All subsequent clinical measurements were performed by the same experienced periodontist using a standardized probing technique, minimizing inter-examiner variability and ensuring internal consistency across the entire dataset.

### Diagnostic criteria

The primary diagnosis was based on the 2017 AAP/EFP classification system, which stages and grades periodontitis according to severity and progression^12^.

Although the AAP/EFP 2017 classification was originally designed for adults, its application in adolescents has been increasingly adopted in recent epidemiological studies to enable comparison across age groups and populations. The criteria provide a unified diagnostic framework and allow early detection of disease stages and grades that may progress into adulthood. To account for potential differences related to age-specific anatomical and inflammatory responses, a sensitivity analysis using the CDC/AAP 2012 surveillance definitions was additionally performed^3^, which categorize individuals as:

- severe periodontitis: ≥2 interproximal sites (not on the same tooth) with CAL ≥ 6 mm and ≥ 1 interproximal site with PPD ≥ 5 mm,
- moderate periodontitis: ≥2 interproximal sites with CAL ≥ 4 mm or ≥ 2 interproximal sites with PPD ≥ 5 mm,
- mild periodontitis: ≥2 interproximal sites with CAL ≥ 3 mm and ≥ 2 interproximal sites with PPD ≥ 4 mm, or 1 site with PPD ≥ 5 mm,
- no periodontitis: when none of the above criteria are met.

### Statistical analysis

Quantitative variables were expressed as means ± standard deviations (SD), while qualitative variables were presented as absolute frequencies and percentages. The normality of quantitative data was assessed using the Kolmogorov–Smirnov test, and homogeneity of variances was checked with Levene’s test. Depending on the number of groups, comparisons of continuous variables were performed using Student’s *t* test (two groups) or one-way ANOVA (≥ three groups). When ANOVA showed significant differences, post hoc pairwise comparisons were conducted using the Bonferroni correction. Categorical variables were compared using the chi-square test or Fisher’s exact test when expected cell counts were < 5. A two-tailed*p*-value < 0.05 was considered statistically significant. All analyses were performed with SPSS software, version 20.0 (SPSS Inc., Chicago, IL, USA).

## Results

The study included 400 students aged between 12 and 18 years, with a mean age of 15.49 ± 1.84 years. The sample consisted of 182 girls (45.5%) and 218 boys (54.5%).The distribution of periodontal status revealed that 63 students (15.7%) had a healthy periodontium, 272 (68.0%) presented gingivitis, and 65 (16.3%) were diagnosed with periodontitis (95% CI: 12.8–20.3). Sex distribution did not differ significantly across groups (p = 0.838,² test). However, mean age varied significantly, with students affected by periodontitis being older than those in the healthy or gingivitis groups (p< 0.001, ANOVA). Periodontal clinical characteristics are summarized in Table 1. A clear gradient was observed from healthy to gingivitis and periodontitis, with significant differences in probing pocket depth (PPD), bleeding on probing (BOP, expressed as % of sites with bleeding), and plaque index (PI) (all p < 0.001). Clinical attachment loss (CAL > 2 mm) and PPD > 3 mm were exclusively detected in the periodontitis group.

Comparison of demographic and clinical parameters among healthy, gingivitis, and periodontitis groups. Variables include mean age, probing pocket depth (PPD), clinical attachment loss (CAL), bleeding on probing (BOP), and plaque index (PI). Values are presented as mean ± standard deviation (SD). § Chi-square test; Ŧ ANOVA. 

**Table 1:** Distribution of periodontal characteristics according to clinical groups (healthy, gingivitis, periodontitis).

**Variable**	**Healthy (n = 63)**	**Gingivitis (n = 272)**	**Periodontitis (n = 65)**	**Total (N = 400)**	**P value**
Gender (Female/Male)	30 / 33	121 / 151	31 / 34	182 / 218	0.838^§^
Mean age ± SD (years)	13.9 ± 1.4	15.4 ± 1.7	17.2 ± 1.0	15.5 ± 1.8	<0.001^Ŧ^
Mean PPD ± SD (mm)	1.94 ± 0.25	2.16 ± 0.32	2.74 ± 0.40	2.22 ± 0.35	<0.001^Ŧ^
Mean PPD > 3 ± SD (mm)	–	–	4.61 ± 0.85	–	–
Mean CAL > 2 ± SD (mm)	–	–	2.92 ± 0.80	–	–
Mean BOP (%)	12 ± 6	81 ± 15	83 ± 18	71 ± 20	<0.001^Ŧ^
Mean PI (%)	78 ± 12	92 ± 10	96 ± 9	91 ± 11	<0.001^Ŧ^

**Fig. 3:**
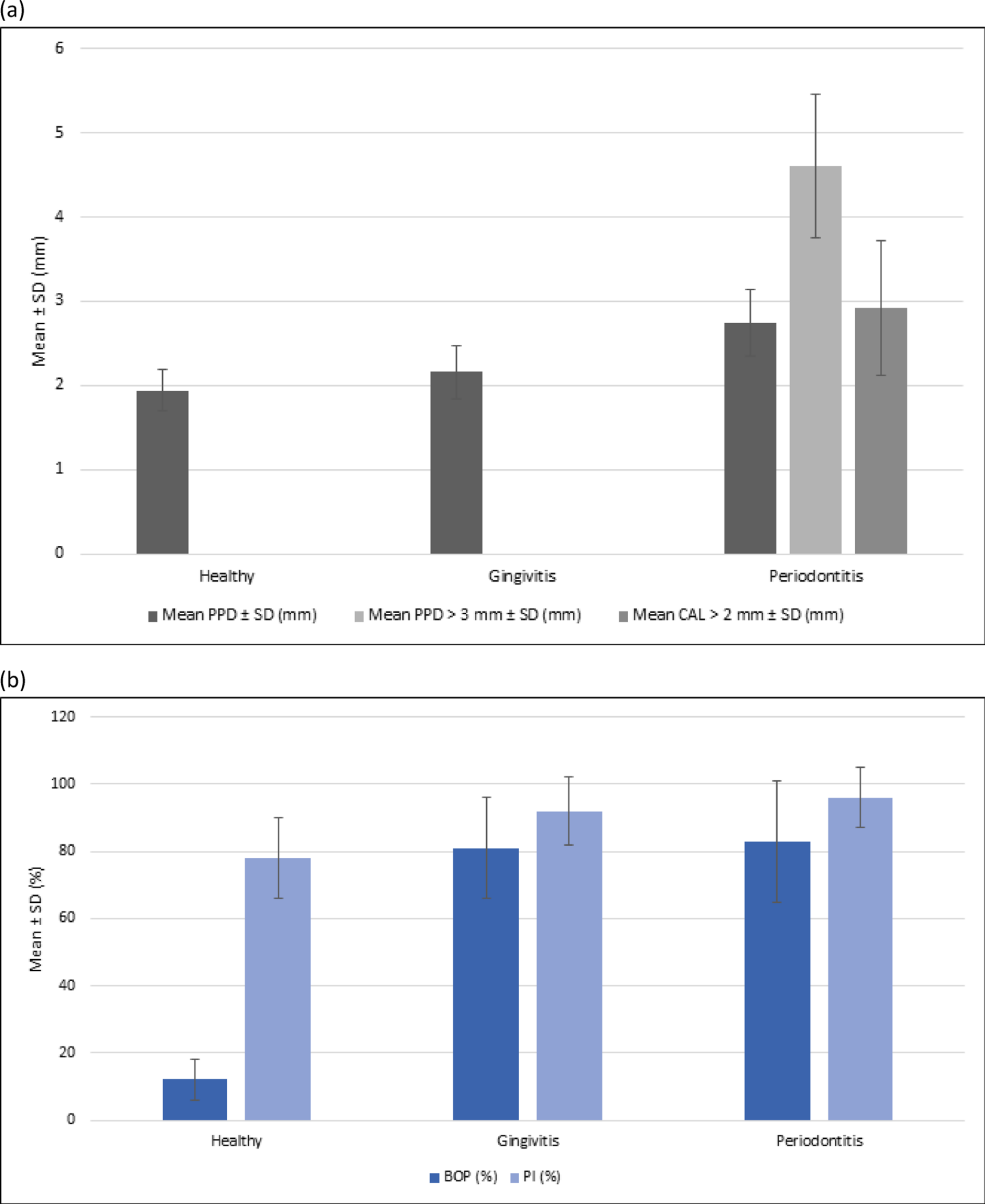
(**a**) Mean probing pocket depth (PPD), proportion of sites with PPD > 3 mm, and mean clinical attachment loss (CAL > 2 mm) across clinical groups. Error bars represent standard deviations. (**b**) Mean bleeding on probing (BOP) and plaque index (PI) across clinical groups. Error bars represent standard deviations.

These trends are further illustrated in Figures 3a and 3b, showing the progressive increase in periodontal destruction (PPD and CAL) and inflammatory parameters (BOP and PI) across the three groups.

Among the 65 adolescents diagnosed with periodontitis, classification according to the 2017 AAP/EFP system showed 26 cases in stage I, 31 in stage II, and 8 in stage III. By grade, 42 were classified as grade B and 23 as grade C. As shown in Table 2, mean values of PPD, CAL > 2 mm, and PPD > 3 mm increased significantly with disease stage (p < 0.001, ANOVA). Significant differences were also observed between grades B and C, particularly in mean pocket depth (p = 0.023, Student’s t test) and mean CAL (p < 0.001, Student’s t test). Given the small number of cases in stage III (n = 8), results for this group should be interpreted with caution.

Distribution of demographic and clinical variables among adolescents with periodontitis stratified by stages (I–III) and grades (B, C) according to the 2017 AAP/EFP classification. Parameters include probing pocket depth (PPD), proportion of sites with PPD> 3 mm, clinical attachment loss (CAL), bleeding on probing (BOP), and plaque index (PI). Values are presented as mean ± SD. § Chi-square or Fisher’s exact test (when expected cell counts < 5); Ŧ ANOVA; Ɨ Student’s t test.

**Table 2:** Periodontal characteristics of students with periodontitis according to stages and grades (AAP/EFP 2017).

**Variable**	**Stage I (n = 26)**	**Stage II (n = 31)**	**Stage III (n = 8)**	**P value**	**Grade B (n = 42)**	**Grade C (n = 23)**	**P value**
Gender (Female/Male)	13 / 13	14 / 17	4 / 4	0.927^§^	20 / 22	11 / 12	0.987^§^
Mean age ± SD (years)	17.3 ± 0.9	17.3 ± 1.0	16.8 ± 1.2	0.356^Ŧ^	17.4 ± 0.8	17.0 ± 1.1	0.176^Ɨ^
Mean PPD ± SD (mm)	2.55 ± 0.45	2.48 ± 0.55	4.37 ± 0.90	<0.001^Ŧ^	2.46 ± 0.5	3.26 ± 0.85	0.023^Ɨ^
Mean PPD > 3 mm ± SD (mm)	4.26 ± 0.50	4.39 ± 0.60	6.63 ± 1.00	<0.001^Ŧ^	4.18 ± 0.45	5.39 ± 095	<0.001^Ɨ^
Mean CAL > 2 mm ± SD (mm)	2.10 ± 0.20	2.90 ± 0.70	6.00 ± 1.00	<0.001^Ŧ^	2.35 ± 0.30	4.17 ± 1.10	<0.001^Ɨ^
Mean BOP (%)	85 ± 12	80 ± 15	87 ± 14	0.707^Ŧ^	84 ± 13	84 ± 13	0.726^Ɨ^
Mean PI (%)	98 ± 5	93 ± 8	99 ± 4	0.445^Ŧ^	95 ± 7	98 ± 5	0.423^Ɨ^

### Sensitivity analysis

When reclassified according to the CDC/AAP 2012 case definitions, the prevalence of periodontitis was markedly lower. Of the 400 adolescents examined, 388 (97.0%) were classified as healthy, 9 (2.3%) as moderate periodontitis, and 3 (0.7%) as severe periodontitis. No cases of mild periodontitis were identified. In contrast, the AAP/EFP 2017 classification identified 63 (15.7%) adolescents with a healthy periodontium, 272 (68.0%) with gingivitis, and 65 (16.3%) with periodontitis. The discrepancy between the two systems illustrates the greater sensitivity of the AAP/EFP 2017 classification for detecting early periodontal changes in adolescents, particularly gingivitis, which is not considered a diagnostic entity in the CDC/AAP 2012 definitions (Table 3).

Distribution of periodontal diagnoses according to the CDC/AAP 2012 and AAP/EFP 2017 classifications. The 2017 system distinguishes healthy, gingivitis, and periodontitis cases with staging and grading, while the 2012 definitions classify only moderate and severe periodontitis, without gingivitis as a diagnostic category.

**Table 3:** Comparison of periodontal status according to AAP/EFP 2017 and CDC/AAP 2012 case definitions.

**Diagnostic**		**CDC/AAP 2012 (n, %)**	**AAP/EFP 2017 (n, %)**
Healthy		388 (97.0%)	63 (15.7%)
Gingivitis		0 (0.0%)	272 (68%)
Periodontitis	Moderate periodontitis	9 (2.3%)	65 (16.3%)
	Severe periodontitis	3 (0.7%)	

## Discussion

 This study investigated the periodontal health status of adolescents aged 12 to 18 years in Rabat, Morocco, using the 2017 AAP/EFP classification system, with a sensitivity analysis based on the CDC/AAP 2012 case definitions. The findings highlight a high burden of gingival inflammation and early periodontal disease in this age group, providing important insights for both clinical practice and public health strategies.

The prevalence of periodontitis in this study was 16.3%, which, although lower than that typically reported in adults, is concerning given the young age of participants. This rate is higher than earlier national reports, such as those by Haubek et al. and Kissa et al^13,14^., but consistent with recent large-scale Moroccan surveys^7,8^. Moreover, 68% of adolescents presented gingivitis, confirming that gingival inflammation is a highly prevalent condition during adolescence. International data similarly indicate that poor oral hygiene practices and inadequate preventive care contribute to early-onset gingival inflammation, which may progress to periodontitis if not addressed^15,16^.

 Several factors may explain the high prevalence of periodontal alterations observed in this study. Adolescence is a critical developmental period marked by hormonal changes, particularly during puberty, which increase the susceptibility of gingival tissues to inflammatory responses even in the presence of modest levels of plaque accumulation^17,18^. Behavioral factors such as inadequate toothbrushing practices, irregular dental visits, and high consumption of sugary foods and beverages further contribute to the persistence of gingival inflammation^19^. In addition, socioeconomic and educational disparities can limit access to preventive care and oral health literacy, reinforcing the accumulation of plaque and calculus. Biological factors may also play a role, as the colonization by periodontopathogenic bacteria such as *Porphyromonas gingivalis* and *Aggregatibacter actinomycetemcomitans* often occurs during adolescence, potentially explaining the presence of stage III or grade C disease in a minority of subjects despite their young age^20,21^. Taken together, these developmental, behavioral, and microbial determinants provide a plausible explanation for the early periodontal alterations observed and emphasize the need for preventive interventions tailored to adolescents.

 The analysis of clinical parameters (PPD, CAL, BOP, PI) demonstrated a clear gradient of deterioration across groups, with significant differences between healthy, gingivitis, and periodontitis categories. These results underscore the central role of gingival inflammation as a precursor to irreversible periodontal damage, corroborating the pathogenic model described by Darveau^22^. The observed increase in disease severity with age within the adolescent group further suggests a cumulative effect, consistent with previous reports in Mediterranean and developing populations^23,24^.

 Applying the AAP/EFP 2017 classification provided a more detailed description of disease stages and grades compared with earlier classifications. Adolescents classified as stage III and grade C exhibited significantly higher levels of attachment loss and probing depth, indicating aggressive disease progression similar to early-onset destructive forms described by Albandar & Rams^25^. These results support the clinical relevance of staging and grading, even in younger populations who are often excluded from adult-based criteria. The distinction between grades B and C was informative, as adolescents in grade C showed markedly worse clinical profiles and would likely require more intensive management^12,26^.

A key strength of this study lies in the comparison between the AAP/EFP 2017 classification and the CDC/AAP 2012 case definitions. The CDC/AAP system identified only 3% of adolescents as having periodontitis, whereas the 2017 criteria detected 16.3% with periodontitis and 68% with gingivitis, leaving only 15.7% classified as healthy. This discrepancy illustrates the limited sensitivity of the 2012 criteria in younger populations and the enhanced ability of the 2017 classification to detect early inflammatory changes. Similar findings have been reported by Morales et al., German et al. and Ortigara et al^4–6^., confirming that the newer framework is more appropriate for epidemiological studies in adolescents.

 From a public health perspective, the high prevalence of gingivitis observed highlights the urgent need for preventive strategies targeting school-aged populations. Poor plaque control and lack of regular dental care remain the main modifiable risk factors. Evidence suggests that school-based oral health promotion and preventive programs are effective in reducing gingival inflammation and plaque accumulation^27^. Adolescence represents a critical period for the establishment of oral hygiene behaviors that can influence long-term oral and systemic health, particularly given the growing recognition of periodontitis as a risk factor for conditions such as diabetes and cardiovascular disease^28,29^.

 Several limitations of this study should be considered. The cross-sectional design prevents causal inference regarding risk factors and disease progression. No socioeconomic, dietary, or microbiological data were collected, limiting the ability to explore determinants of periodontal health in depth. Furthermore, only one examiner performed the clinical assessments, and no formal calibration was carried out. Although this ensured consistency in measurement, the lack of reproducibility testing is acknowledged as a limitation. Future epidemiological research in adolescents should incorporate examiner calibration, longitudinal designs, and a broader set of biological and behavioral variables to better capture risk patterns and disease dynamics.

## Conclusion

 This study demonstrates that periodontal diseases, particularly gingivitis, are highly prevalent among Moroccan adolescents, with a substantial proportion already showing early signs of periodontitis. The comparison between the CDC/AAP 2012 and AAP/EFP 2017 case definitions highlights the greater sensitivity of the latter in detecting early inflammatory and destructive changes, supporting its applicability in adolescent populations. From a public health perspective, these findings call for the implementation of school-based oral health education programs, routine periodontal screening in adolescents, and training of school health personnel to promote early detection and prevention. Integrating these measures into existing national oral health strategies could substantially reduce the long-term burden of periodontal disease. Future research should aim to validate the 2017 AAP/EFP classification in younger age groups, explore microbiological and behavioral risk factors associated with early periodontal breakdown, and evaluate the effectiveness of targeted preventive interventions in school settings through longitudinal studies.

## Data Availability

The datasets used and/or analyzed during the current study are available from the corresponding author on reasonable request.
